# *Candida albicans* Scavenges Host Zinc via Pra1 during Endothelial Invasion

**DOI:** 10.1371/journal.ppat.1002777

**Published:** 2012-06-28

**Authors:** Francesco Citiulo, Ilse D. Jacobsen, Pedro Miramón, Lydia Schild, Sascha Brunke, Peter Zipfel, Matthias Brock, Bernhard Hube, Duncan Wilson

**Affiliations:** 1 Department of Microbial Pathogenicity Mechanisms, Leibniz Institute for Natural Product Research and Infection Biology, Hans Knöll Institute (HKI), Jena, Germany; 2 Center for Sepsis Control and Care, Jena University Hospital, Jena, Germany; 3 Department of Infection Biology, Leibniz Institute for Natural Product Research and Infection Biology, Hans Knöll Institute (HKI), Jena, Germany; 4 Friedrich Schiller University, Jena, Germany; 5 Microbial Biochemistry and Physiology, Leibniz Institute for Natural Product Research and Infection Biology, Hans Knöll Institute (HKI), Jena, Germany; Carnegie Mellon University, United States of America

## Abstract

The ability of pathogenic microorganisms to assimilate essential nutrients from their hosts is critical for pathogenesis. Here we report endothelial zinc sequestration by the major human fungal pathogen, *Candida albicans*. We hypothesised that, analogous to siderophore-mediated iron acquisition, *C. albicans* utilises an extracellular zinc scavenger for acquiring this essential metal. We postulated that such a “zincophore” system would consist of a secreted factor with zinc-binding properties, which can specifically reassociate with the fungal cell surface. *In silico* analysis of the *C. albicans* secretome for proteins with zinc binding motifs identified the pH-regulated antigen 1 (Pra1). Three-dimensional modelling of Pra1 indicated the presence of at least two zinc coordination sites. Indeed, recombinantly expressed Pra1 exhibited zinc binding properties *in vitro*. Deletion of *PRA1* in *C. albicans* prevented fungal sequestration and utilisation of host zinc, and specifically blocked host cell damage in the absence of exogenous zinc. Phylogenetic analysis revealed that *PRA1* arose in an ancient fungal lineage and developed synteny with *ZRT1* (encoding a zinc transporter) before divergence of the Ascomycota and Basidiomycota. Structural modelling indicated physical interaction between Pra1 and Zrt1 and we confirmed this experimentally by demonstrating that Zrt1 was essential for binding of soluble Pra1 to the cell surface of *C. albicans*. Therefore, we have identified a novel metal acquisition system consisting of a secreted zinc scavenger (“zincophore”), which reassociates with the fungal cell. Furthermore, functional similarities with phylogenetically unrelated prokaryotic systems indicate that syntenic zinc acquisition loci have been independently selected during evolution.

## Author Summary

The capacity of disease-causing microbes to acquire nutrients from their host is one of the most fundamental aspects of infection. Host organisms therefore restrict microbial access to certain key nutrients in a process known as nutritional immunity. Recently, it was found that infected vertebrates sequester zinc from invading microorganisms to control infection. Therefore, the mechanisms of microbial zinc acquisition represent potential virulence attributes. Here we report the molecular mechanism of host-derived zinc acquisition by the major human fungal pathogen, *Candida albicans*. We show that *C. albicans* utilises a secreted protein, the pH-regulated antigen 1 (Pra1), to bind zinc from its environment. Pra1 then reassociates with the fungal cell via a syntenically encoded (genetically-linked) membrane transporter (Zrt1) to acquire this essential metal. Deletion of *PRA1* prevented utilisation of host zinc and damage of host cells in the absence of exogenous zinc. Finally, we demonstrate that this zinc-scavenging locus arose in an ancient fungal lineage and remains conserved in many contemporary species. Syntenically arranged zinc acquisition systems have evolved independently in the fungal and bacterial kingdoms, suggesting that such an arrangement is evolutionary beneficial for microorganisms.

## Introduction

Assimilation of essential nutrients by pathogenic microorganisms from their host environment is one of the most fundamental aspects of infection. Host organisms therefore restrict microbial access to certain key nutrients in a process termed nutritional immunity. The mechanisms of iron sequestration, together with the strategies that successful pathogens employ to overcome this restriction have been extensively studied [1]. Zinc is the second most abundant trace metal in vertebrates and an important cofactor for around 9% of eukaryotic proteins [2]. However, unlike iron, the microbial mechanisms of zinc acquisition are not as well understood. Recently, Corbin and coworkers demonstrated that infected mice actively sequester zinc from invading bacteria [3]; therefore, the scope of nutritional immunity has expanded beyond iron [4] and the mechanisms of microbial zinc acquisition represent potential virulence factors.

*Candida albicans* is one of the few fungal species of the normal human microbiome. Although typically a commensal of the oral cavity, gastrointestinal and urinogenitary tracts, *C. albicans* is also an extremely frequent cause of superficial infections such as vaginitis. Moreover, common iatrogenic procedures, such as gastrointestinal surgery, implantation of a central venous catheter or antibiotic treatment are major risk factors for disseminated candidiasis. This form of systemic candidiasis is now the third most common cause of nosocomial bloodstream infections and the mortality of severe sepsis caused by *Candida* species is over 50% [5].

*C. albicans* virulence relies on a number of factors, including morphological plasticity, the expression of adhesins and invasins, robust stress responses, immune evasion, metabolic flexibility and nutrient acquisition [6], [7], [8]. A number of studies have focused on how *C. albicans* assimilates iron [9]; however the mechanisms of zinc acquisition by pathogenic fungi are poorly understood.

In the current study we sought to elucidate the mechanism of *C. albicans* zinc acquisition from host cells. We found that *C. albicans* secretes a scavenger protein (a “zincophore”), Pra1, which sequesters zinc from host cells and re-associates with the fungus via a co-expressed zinc transporter, Zrt1. Furthermore, we show that syntenic zinc acquisition loci are conserved in many fungal species with functional similarities to bacterial ABC transport systems.

## Results

### Invasive *C. albicans* hyphae sequester host zinc

Our first objective was to determine whether *C. albicans* can acquire zinc from host cells. During colonisation of the oral mucosa, vagina or gastrointestinal tract, *C. albicans* coexists with other members of the microbial flora and is exposed to a complex milieu of nutrients. However, following infection of otherwise sterile body sites, the only nutrients available to the fungus are from host cells, tissues or extracellular matrix and fluids. We therefore decided to focus on a specific stage of systemic candidiasis: interaction with human endothelial cells.

We first created zinc-depleted cell culture medium by treating Dulbecco's Modified Eagle's Medium (DMEM) with CHELEX-100 beads and then reconstituting all metals, with the exception of zinc, to their original concentrations (DMEM-Zn). To ensure that this zinc restriction did not adversely affect the endothelia, monolayers of HUVECs were incubated with DMEM or DMEM-Zn for 24 h and host cell damage was assayed by measuring release of lactate dehydrogenase (LDH). Zinc depletion did not result in increased LDH release, demonstrating that removal of this metal was not cytotoxic over the investigated time frame (data not shown).

**Figure 1:**
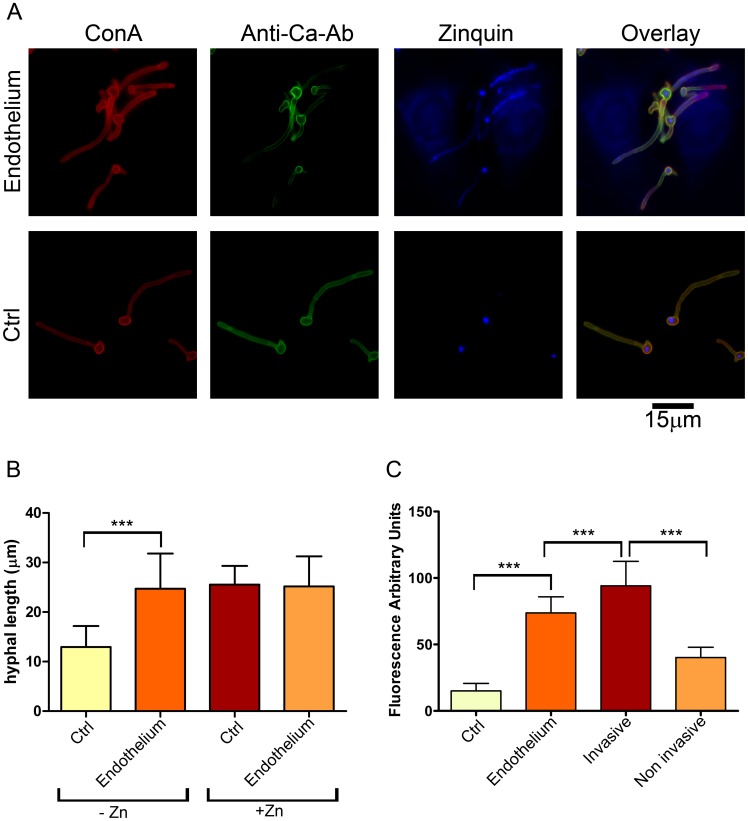
Invasive *C. albicans* hyphae sequester endothelial zinc. (**A**) Endothelial monolayers were infected with wild type *C. albicans* (M134) for 3.5 h in zinc-free medium. As a control (Ctrl), *C. albicans* was incubated under identical conditions in the absence of endothelium. Concanavalin A (ConA) stains the entire fungus red; anti-*Candida* antibody (Anti-Ca-Ab) stains only the extracellular (non-invasive) part of the fungus green; zinquin stains zinc blue. Note that the invasive (ConA^+^/Anti-Ca-Ab^−^) sections of *C. albicans* hyphae stain positive for zinquin. (**B**) Quantification of hyphal length of *C. albicans* incubated for 3.5 h in the absence or presence of endothelia, either with or without zinc supplementation. (**C**) Quantification of zinquin intensity of *C. albicans* hyphae incubated in zinc-free medium in the absence (Ctrl) or presence of Endothelium; additionally, zinquin intensity of intracellular (invasive) and extracellular (non invasive) hyphae was determined. Experiment was performed twice in triplicate. Asterisks indicate significance (*P*<0.001) by Student's t-test.

To investigate whether *C. albicans* can utilise host zinc, zinc-starved yeast cells were incubated either with or without endothelial monolayers in zinc-depleted medium for 3.5 h and hyphal length determined as a measure of growth (**Figure 1A**). *C. albicans* formed significantly longer hyphae in the presence of endothelial cells (**Figure 1B**). This was not due to enhanced filamentation upon contact with endothelial cells *per se*, as supplementation of the medium with zinc restored growth. (**Figure 1B**). These observations suggested that *C. albicans* can use zinc from host cells.

To investigate this further, we treated the cells with zinquin, a specific dye which fluorescently labels zinc. As shown in **Figure 1A**, the mother cells of hyphae (both in the presence and absence of endothelia) stained positive for zinquin, suggesting that the inoculated yeast cells carried over some zinc from the preculture. Emergent hyphae, on the other hand, exhibited punctate zinquin staining only in the presence of endothelia (**Figure 1A**). Indeed, the fluorescence intensity of endothelial-associated hyphae was around 5-fold higher than hyphae grown without endothelia (**Figure 1C**).

As the only other zinc available to *C. albicans* is from host cells, we reasoned that fungal invasion of the endothelia may facilitate zinc acquisition. We therefore performed differential fluorescent staining [10] to directly visualise and discriminate invading and non-invading fungal elements. As predicted, the invading hyphal elements bound zinc at significantly higher levels than the non-invading sections (**Figure 1C**).

Together these data demonstrate that invading *C. albicans* hyphae are able to sequester zinc from endothelial cells.

### The pH regulated antigen, Pra1, is a zinc-binding protein

The mechanisms of microbial iron acquisition are well documented [1]. Arguably one of the most widespread strategies is the utilisation of siderophores – small secreted molecules which chelate iron with high affinity and can return to a microbial cell to deliver their iron load. Given the evolutionary success of siderophores, we hypothesised that *C. albicans* may employ an analogous system, secreting a metal-binding molecule to capture zinc. We reasoned that proteins constitute promising candidates for such a function, as around 9% of eukaryotic proteins can bind zinc [2].

**Figure 2:**
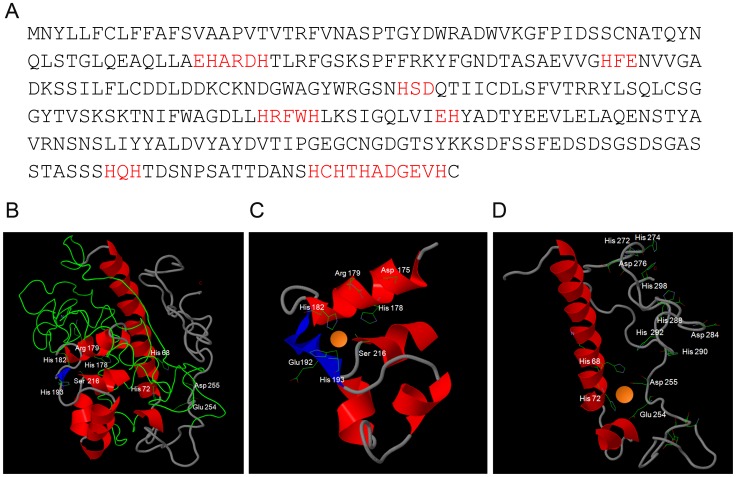
*In silico* prediction of Pra1-zinc binding. (**A**) Primary amino acid sequence of Pra1 (*Candida* Genome Database) with zinc-binding motifs in red. (**B**) Three-dimensional model of Pra1 built with Phyre^2^. (**C**) and (**D**) close-up of predicted zinc coordination sites. Note the presence of arginine, rather than glutamic acid at residue 179 (**C**) and that the C-terminal tail of Pra1 has multiple additional potential zinc binding histidine residues (**D**).

The amino acid sequences of all 55 confirmed and predicted *C. albicans* secreted proteins (GO term, extracellular region – *Candida* Genome Database) were analysed using protein-pattern-find for the presence of zinc binding motifs [11]. Of the *C. albicans* secretome, only the pH regulated antigen 1, encoded by *PRA1*, contained multiple zinc-binding motifs (highlighted in red **Figure 2A**).

Three-dimensional modelling of the Pra1 sequence predicted a best-fit with Deuterolysin of *Aspergillus oryzae*. The three-dimensional structure is shown in **Figure 2B**. Pra1 was predicted to host two zinc-binding coordination sites (**Figure 2C & D**) with additional multiple zinc binding histidine residues in the tail (**Figure 2D**). Deuterolysin is an M35 metalloprotease [12], characterised by an HEXXH motif at the catalytic site. However, manual analysis of the Pra1 sequence revealed degeneration of this motif with a glutamic acid to arginine substitution in Pra1 (**Figure 2A & C**). Indeed, when we measured the proteolytic activity of purified Pra1 against the metalloprotease substrate casein, Pra1 did not exhibit protease activity, in contrast to a positive control (thermolysin), (**Figure S1**). From these analyses, we conclude that Pra1 has lost (or has never had) protease activity, but may possess zinc binding capacity.

**Figure 3:**
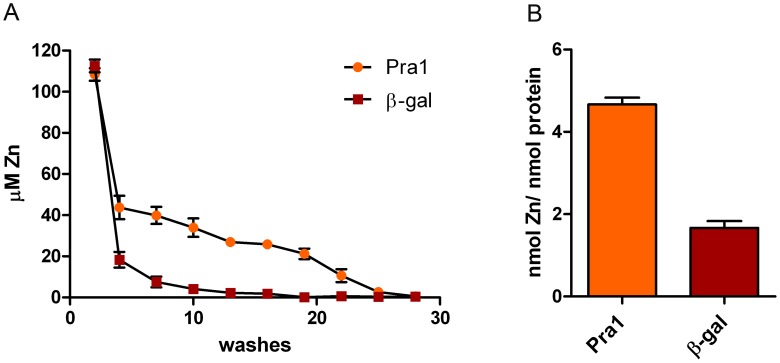
Pra1 is a zinc binding protein. (**A**) Recombinant Pra1 or β-galactosidase were pre-incubated with zinc, loaded on 10 kDa microspin columns and sequentially washed with Hs buffer. The zinc content of each flow-through was determined by PAR assay. (**B**) The fully washed proteins were proteolytically digested and released zinc measured by PAR assays. Experiment was performed 3 times in duplicate.

To test this hypothesis, we directly measured the zinc binding capacity of recombinant purified His-tagged Pra1 [13]. His-tagged β galactosidase [14] was included as a control to account for the metal binding properties of the His tag. Zinc-loaded protein samples were sequentially washed on 10 kDa microspin columns and the zinc content of each flow-through was measured using a PAR assay. Following 10 washes of β galactosidase, zinc was no longer detectable in the flow-through. In contrast, we continued to measure zinc in the Pra1 flow-through until the 23^rd^ wash (**Figure 3A**), suggesting that Pra1 can loosely bind relatively large amounts of zinc.

The fully washed protein samples were then digested with proteinase K and assayed for zinc release. As shown in **Figure 3B**, digested Pra1 released 4.6 moles zinc per mole protein. In contrast, His-tagged β galactosidase released only 1.8 moles zinc per mole protein. Therefore, in agreement with our modelling approach (**Figure 2**), Pra1 can tightly bind approximately three zinc atoms. We also tested the ability of Pra1 to bind iron, calcium, copper and manganese. We observed no binding of iron, calcium or manganese and only moderate binding of copper (data not shown). We therefore conclude that zinc is the dominant metal-substrate of Pra1.

### Pra1 mediates endothelial damage by scavenging host zinc

**Figure 4:**
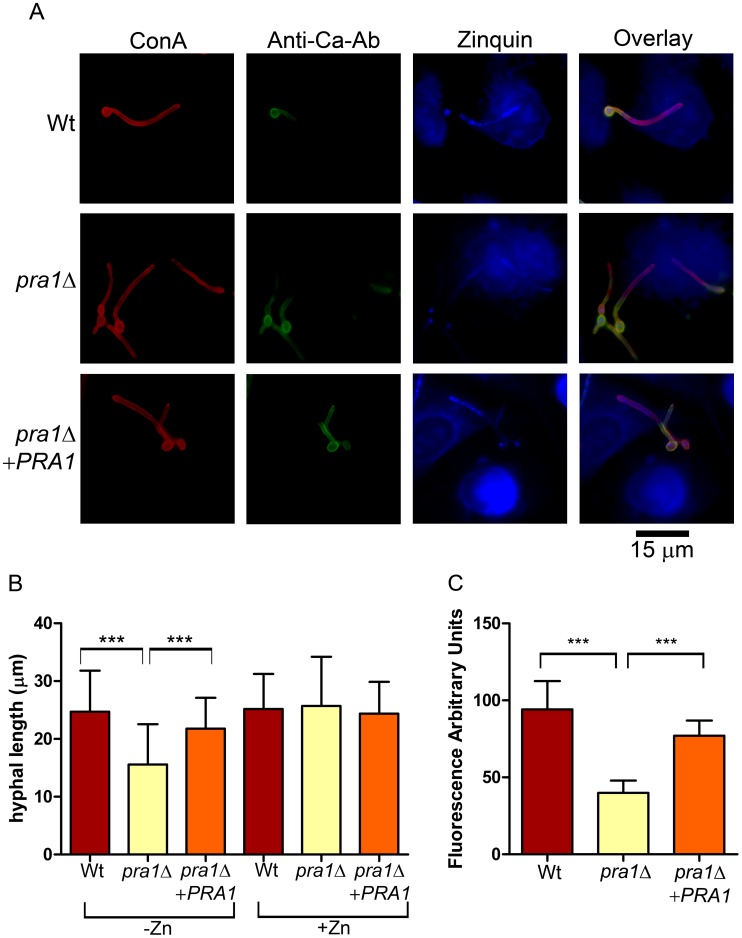
Deletion of *PRA1* precludes host zinc sequestration. (**A**) Endothelial monolayers were infected with wild type (M134), *pra1*Δ (M1809) or *pra1*Δ+*PRA1* (M1785) *C. albicans* strains for 3.5 h in zinc-free medium. Concanavalin A (ConA) stains the entire fungus red; anti-*Candida* antibody (Anti-Ca-Ab) stains only the extracellular (non-invasive) part of the fungus green; zinquin stains zinc blue. Note that *pra1*Δ does not accumulate zinc from the host cell. (**B**) Quantification of hyphal length of *C. albicans* in association with endothelia in either zinc free medium (−Zn) or with supplementation with 20 µM zinc (+Zn). (**C**) Quantification of zinquin fluorescence intensity per area of the invasive (intracellular) portion of *C. albicans* hyphae. Experiment was performed twice in triplicate. Asterisks indicate significance (*P*<0.001) by Student's t-test.

Having established that *C. albicans* hyphae can sequester host zinc and that Pra1 is able to effectively bind this metal *in vitro*, we anticipated that Pra1 is responsible for zinc sequestration during invasive growth. *C. albicans* zinc-starved wild type, *pra1*Δ and *pra1*Δ+*PRA1* strains were used to infect endothelial cells in the absence or presence of exogenous zinc. All strains formed hyphae on endothelia (**Figure 4A**). However, in the absence of exogenous zinc, *pra1*Δ hyphae were significantly shorter than the wild type (**Figure 4B**). Normal hyphal growth of the *pra1*Δ mutant was restored either by genetic complementation with a single copy of *PRA1* or by reconstituting the medium with 20 µM zinc (**Figure 4B**). Differential fluorescence staining revealed that, despite the reduced hyphal length of *pra1*Δ in the absence of zinc, the actual percentage of invading hyphae were similar amongst strains (data not shown). Therefore, the growth defect of *pra1*Δ was not due to a general inability to physically access intra-endothelial zinc. To determine whether Pra1 binds zinc within endothelia, we stained with zinquin to visualise localisation of the metal. In contrast to wild type and *pra1*Δ+*PRA1* strains, punctate zinquin staining was not observed on the hyphae of the *pra1*Δ mutant (**Figure 4A**) and the fluorescence intensity of *pra1*Δ hyphae was significantly lower (**Figure 4C**). Therefore, Pra1 is required for zinc sequestration within host endothelial cells.

**Figure 5:**
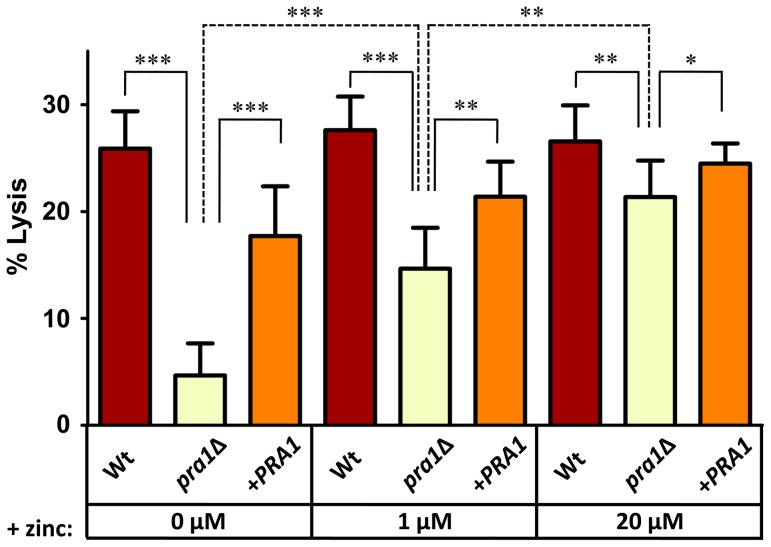
Endothelial damage is zinc-dependent in the absence of *PRA1*. Endothelial monolayers were infected with wild type (M134), *pra1*Δ (M1809) or *pra1*Δ+*PRA1* (M1785) *C. albicans* in zinc-free medium with indicated zinc-supplementation. Following 24 h co-incubation, endothelial damage was assessed by measuring the release of lactate dehydrogenase. Experiment was performed 3 times in triplicate. Asterisks indicate significance by Student's t-test: * <0.05; ** <0.01; *** <0.001.

To examine the impact of Pra1-mediated zinc scavenging on the pathogenicity of *C. albicans*, we co-incubated different zinc-starved *C. albicans* strains with endothelial monolayers in the presence of varying concentrations of exogenous zinc for 24 h, and assayed host cell damage by measuring release of lactate dehydrogenase (LDH). The zinc content of the medium did not influence endothelial damage caused by wild type cells, suggesting that *C. albicans* can efficiently assimilate this micronutrient directly from the endothelia. In contrast, *pra1*Δ caused very little damage to host cells in the absence of exogenous zinc. However, reconstitution of the medium with zinc resulted in near wild type levels of endothelial damage by *pra1*Δ (**Figure 5**). Therefore Pra1 is specifically required for host cell damage in the absence of free zinc.

### *PRA1* expression is regulated by pH and zinc availability

**Figure 6:**
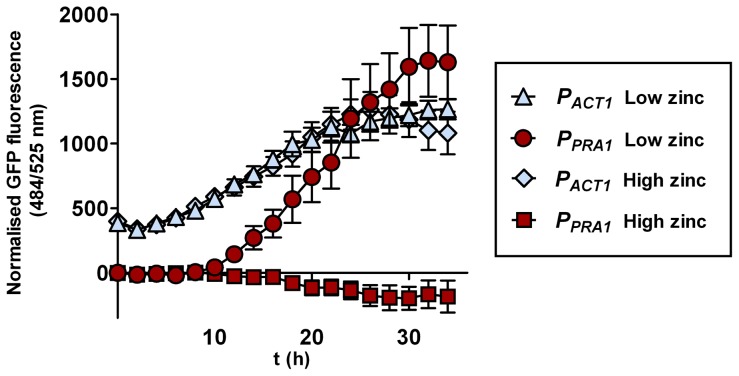
*PRA1* expression is zinc regulated. *C. albicans* harbouring GFP under control of either the *ACT1* (*P_ACT1_*-GFP, M135) or *PRA1* (*P_PRA1_*-GFP, M1522) promoter were incubated in Lee's medium, pH 7.4 either with or without supplementation with (100 µM) zinc. GFP fluorescence was determined at indicated time points and normalised against a *C. albicans* strain harbouring an empty vector. Note that the addition of zinc fully suppresses expression from the *PRA1* promoter. Experiment was performed three times in triplicate.

*PRA1* is expressed at alkaline, but not acidic pH [15]. As *PRA1* is specifically required for hyphal extension and endothelial damage under zinc limitation and encodes a zinc scavenger, we predicted that this gene should be responsive to environmental zinc levels. We therefore grew *C. albicans* harbouring a codon optimised GFP (green fluorescent protein) [16] under the control of the *PRA1* promoter (*P_PRA1_*) in Lee's medium buffered to either pH 5.5 or pH 7.4. As expected [15], *P_PRA1_* drove robust GFP expression at alkaline, but not at acidic pH. The addition of exogenous zinc (100 µM) to Lee's pH 7.4 fully blocked *P_PRA1_*-GFP expression (**Figure 6**). Zinc depletion at pH 5.5, on the other hand, did not induce *PRA1* expression, indicating that *PRA1* is strongly repressed at acidic pH. We also tested the effect of other metals (iron, copper and manganese at 100 µM) on *PRA1* expression at alkaline pH. In contrast to high levels of zinc, these other transition metals did not repress *PRA1* expression (data not shown). Therefore, *PRA1* expression is regulated by both environmental pH and zinc.

### A conserved zinc acquisition locus

As noted by Nobile and colleagues [17], *PRA1* shares its upstream intergenic region with *ZRT1*, which encodes a predicted high affinity zinc transporter. Moreover, these two genes are transcriptionally co-regulated, belonging to the same transcription module [18] and exhibit similar expression patterns during invasion of oral epithelial cells [19] and liver tissue [20].

**Figure 7:**
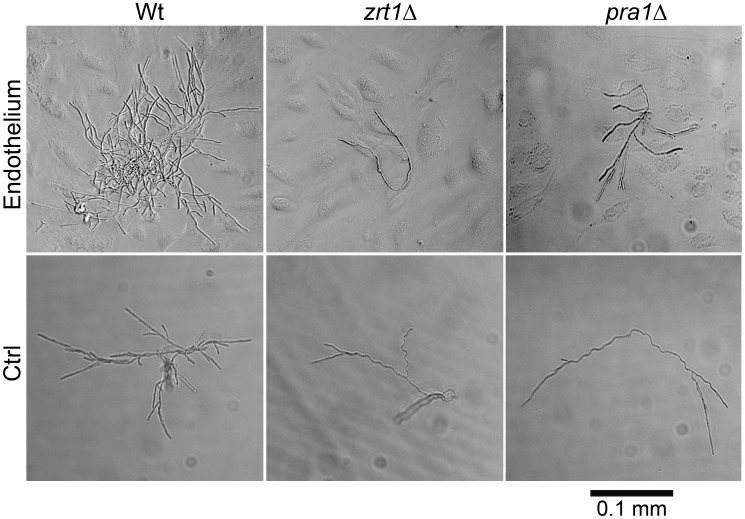
*ZRT1* and *PRA1* are required for microcolony development on endothelia in the absence of exogenous zinc. Single cells of *C. albicans* wild type (M1477), *zrt1*Δ (M2006) or *pra1*Δ (M2008) were incubated for 16 h in zinc-depleted cell culture medium on endothelial monolayers (Endothelium) or on plastic (Ctrl). Experiment was performed three times. representative images are shown. Note that only wild type cells were able to assimilate sufficient zinc for microcolony development.

We postulated that *PRA1*-*ZRT1* represents a zinc acquisition locus. We therefore deleted *ZRT1* in *C. albicans* and tested the growth behaviour of the resultant mutant in zinc depleted medium. The *zrt1*Δ mutant grew poorly in the absence of exogenous zinc (**Figure S2**). Growth was restored to wild type levels either by genetic complementation with a single copy of *ZRT1* or by supplementing the medium with zinc. Next, to investigate whether Zrt1 was also required for assimilation of zinc from host cells, we assayed fungal growth with or without endothelial monolayers in zinc-depleted medium. Under control conditions without endothelial cells, wild type, *zrt1*Δ or *pra1*Δ strains grew poorly (**Figure 7**). In the presence of endothelia, wild type cells formed larger, regularly shaped micro-colonies. In contrast, growth of *zrt1*Δ and *pra1*Δ was not enhanced by the presence of endothelia (**Figure 7**). Supplementation of the medium with zinc resulted in large regularly shaped micro-colonies for all strains, irrespective of the presence of endothelia (**Figure S3**). This suggests that *C. albicans* employs both Pra1 and Zrt1 for efficient assimilation of zinc from host cells.

We conclude that, in agreement with phylogenetic evidence and *ZRT1* over-expression analysis [17], [21], Zrt1 is indeed a zinc transporter and that *PRA1*-*ZRT1* represents a zinc acquisition locus.

Amich and co-workers [22] have recently reported that *A. fumigatus zrfC* (encoding a high affinity zinc transporter) shares its upstream intergenic region with *aspf2* (encoding a fibrinogen binding allergen). The authors also demonstrated that these genes are co-regulated and required for growth under zinc limitation.

Interestingly, *C. albicans* Pra1 and *A. fumigatus* Aspf2 have similar properties [17], [23]. Indeed, upon sequence alignment, we observed 43% identity between the Pra1 and Aspf2 amino acid sequences and 48% identity between Zrt1 and ZrfC sequences. As *A. fumigatus aspf2*-*zrfC* and *C. albicans PRA1*-*ZRT1* are syntenic and all four genes required for efficient zinc assimilation, it would appear that the zinc acquisition locus is conserved in these two species. We therefore investigated whether this locus is also conserved in other species.

BLASTp analysis with the *C. albicans* Pra1 amino acid sequence as query identified orthologues in a diverse, yet limited, number of fungal species (**Figure S4**).

**Figure 8:**
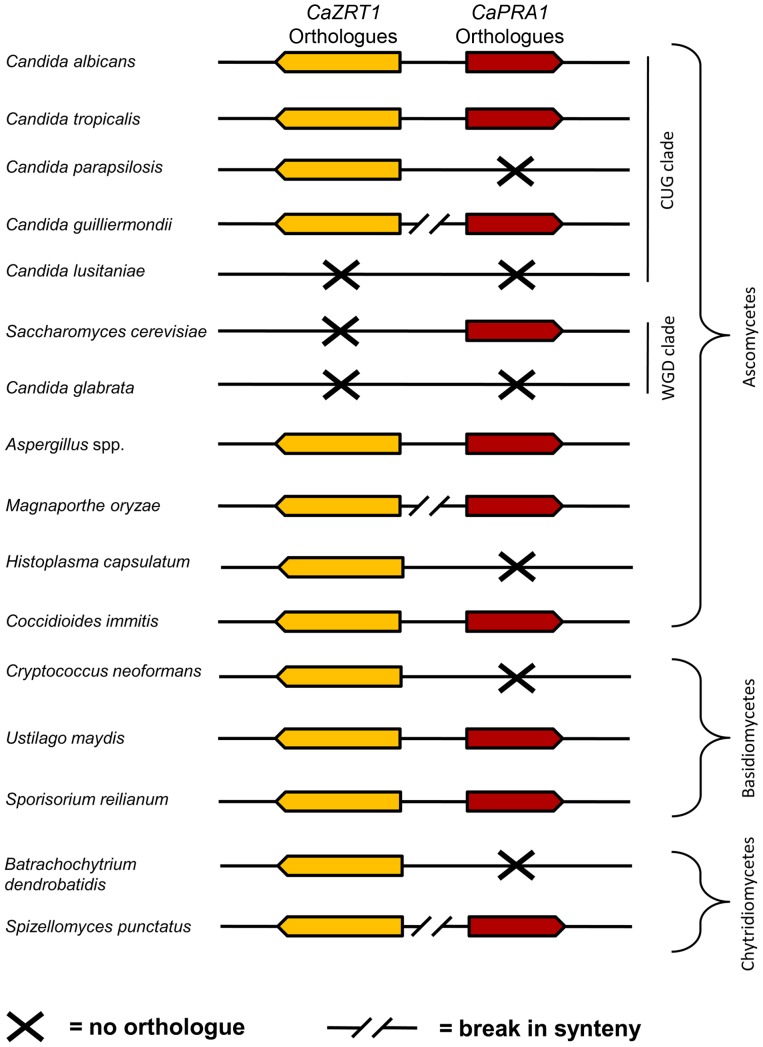
Synteny of genes encoding zinc transporters and zinc-binding proteins. Genomic arrangement of *C. albicans ZRT1* and *PRA1* orthologues in selected fungal species. Only direct orthologues of *C. albicans ZRT1* are shown (CaZrt1-cluster, see Figure S5). Note that synteny is conserved between distantly related species (e.g. *C. albicans* and *U. maydis*) but has broken down in more closely related species (e.g. *C. albicans* and *C. parapsilosis*).

In agreement with Amich *et al.* [22], we observed synteny of the *A. fumigatus* orthologues of *PRA1* (*aspf2*) and *ZRT1* (*zrfC*). Indeed, *PRA1* and *ZRT1* orthologues were syntenic in all sequenced *Aspergillus* species. Within the *Candida* (CUG) clade, *ZRT1*-*PRA1* synteny was conserved in some, but not all species (**Figure 8**). Interestingly, in the distantly related Basidiomycetes, *Ustilago maydis* and *Sporisorium reilianum*, *PRA1* and *ZRT1* orthologues also share their upstream intergenic region. We did not detect *PRA1* orthologues in Zygomycete or Microsporidia species. However, the basal Chytrid *Spizellomyces punctatus* does have a *PRA1* orthologue, although it does not share synteny with a *ZRT1* orthologue. Together, these observations suggest that *PRA1* arose in an ancient fungal lineage and that its syntenic arrangement with *ZRT1* occurred before divergence of the Basidiomycota and Ascomycota – an event which occurred at least 452 million years ago [24]. Subsequently, however, *PRA1* has been lost multiple times. Indeed, most contemporary fungal clades encompass species both with and without *PRA1* orthologues (**Figure 8**).

*PRA1* orthologues are strictly specific to fungi. *ZRT1*, on the other hand, belongs to the ZIP (ZRT/IRT-like protein) family of transporters, an ancient family found in both eukaryotes and prokaryotes [25]. Unlike their fungal counterparts, however, bacterial ZIP transporters are encoded at loci unlinked to other known zinc acquisition system components. Rather, bacteria possess ZnuABC systems for high affinity zinc uptake [26]. These ABC transporters generally consist of an ATP-binding protein (ZnuC), a permease (ZnuB) and a plasma membrane or periplasmic substrate-binding protein (ZnuA), encoded at the same locus.

Significantly, ZnuB permeases are not phylogenetically related to the ZIP family of zinc transporters and the ZnuA zinc binding proteins are unrelated to fungal Pra1 orthologues.

### Zrt1 mediates Pra1 re-association

In Gram negative bacterial ABC transport systems, the soluble binding protein (ZnuA) associates with its substrate in the periplasm for delivery to the cognate membrane permease (ZnuB) [27]. As soluble Pra1 has been shown to reassociate with the *C. albicans* cell surface [13], we hypothesised that, analogous to the bacterial ABC systems, Zrt1 and Pra1 may physically interact to facilitate zinc uptake.

**Figure 9:**
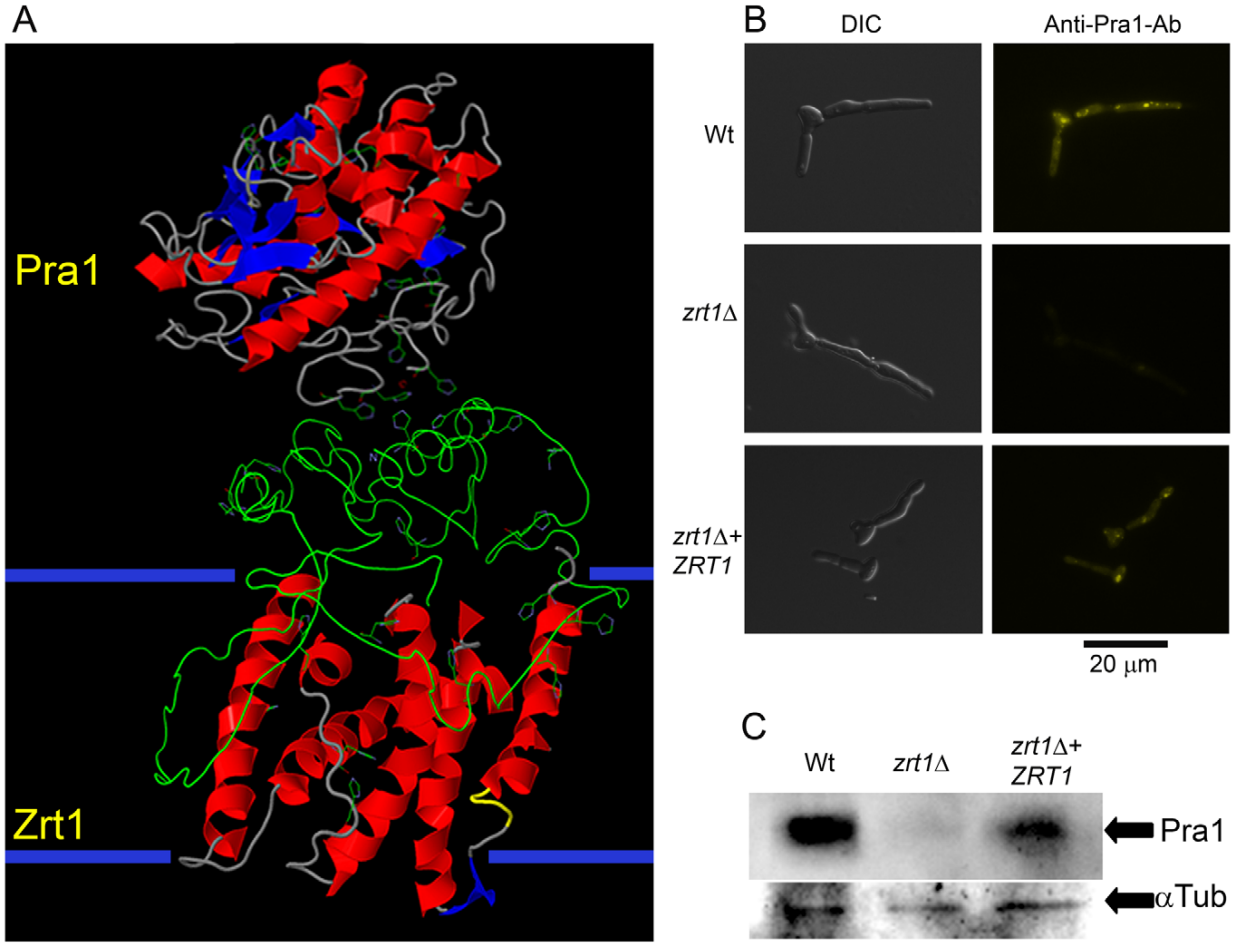
Zrt1 is required for reassociation of soluble Pra1 to the fungal cell. (**A**) Predicted three-dimensional structures and interaction of Pra1 and Zrt1. The large extracellular domain of Zrt1 represents the N-terminal tail from amino acids 1–183. (**B**) *C. albicans* wild type (M1477), *zrt1*Δ (M2006) and *zrt1*Δ+*ZRT1* (M2010) were exposed to recombinant His-tagged Pra1 and binding visualised with a fluorescently conjugated anti-His antibody (experiment was performed twice in triplicate); (**C**) additionally, binding of rPra1 was assessed by Western Blot hybridisation (experiment was performed twice). Note that deletion of *ZRT1* precludes reassociation of Pra1 with the fungal cell.

Three-dimensional modelling of Zrt1 and molecular docking algorithms predicted an interaction between Zrt1 with Pra1 (**Figure 9A**). To test this prediction experimentally, we exposed *C. albicans* to recombinant His-tagged Pra1 and visualised binding with a fluorescently conjugated anti-His antibody. We detected association of rPra1 to wild type, but not *zrt1*Δ cells. Complementation of the *zrt1*Δ mutant with a single copy of *ZRT1* restored Pra1 binding (**Figure 9B**). In a parallel approach, we exposed *C. albicans* to recombinant His-tagged Pra1 and assayed binding via Western blot analysis. As shown in **Figure 9C**, the presence of Pra1 was clearly detectable in protein extract from wild type, but not *zrt1*Δ cells. Complementation with *ZRT1* restored Pra1 binding albeit to a slightly lesser extent than the wild type. Together these data demonstrate that Zrt1 is essential for reassociation of soluble Pra1 to *C. albicans* cells.

## Discussion

Whilst iron acquisition is an established virulence determinant of many microbial pathogens, the role of zinc, and other essential transition metals, in pathogenicity, has historically received less attention. Recently, however, it has been demonstrated that *Staphylococcus aureus*-infected mice can actively sequester zinc and manganese from invasive bacterial cells. Therefore the scope of nutritional immunity has expanded beyond iron [4]. Moreover, a number of bacterial zinc transport systems have now been characterised and shown to be involved in virulence [28], [29]. In contrast, the mechanisms employed by human pathogenic fungi to acquire zinc from their hosts have remained unclear.

Pra1, was first identified as a fibrinogen-binding mannoprotein [23], [30], and then, based on expression studies, designated pH-regulated antigen 1 [15]. Pra1 is notable for its complex relationship with innate immunity [31]. On the one hand, Pra1 expression can increase *C. albicans* recognition by neutrophils, as it serves as the major ligand for the leukocyte integrin α_M_β_2_ [32]. Indeed, deletion of *PRA1* can dampen neutrophil activation [33], protect *C. albicans* from killing by leukocytes and actually increases the virulence of *C. albicans* in some animal models [34]. In this context, it is noteworthy that *PRA1* expression is 29-fold down-regulated during incubation with human blood [35]. On the other hand, expression of Pra1 benefits *C. albicans* by recruiting complement inhibitors (factor H, factor H-like protein-1 and C4b-binding protein) to the fungal cell surface and by binding C3, thus preventing its cleavage to C3a and C3b [13], [36], [37].

**Figure 10:**
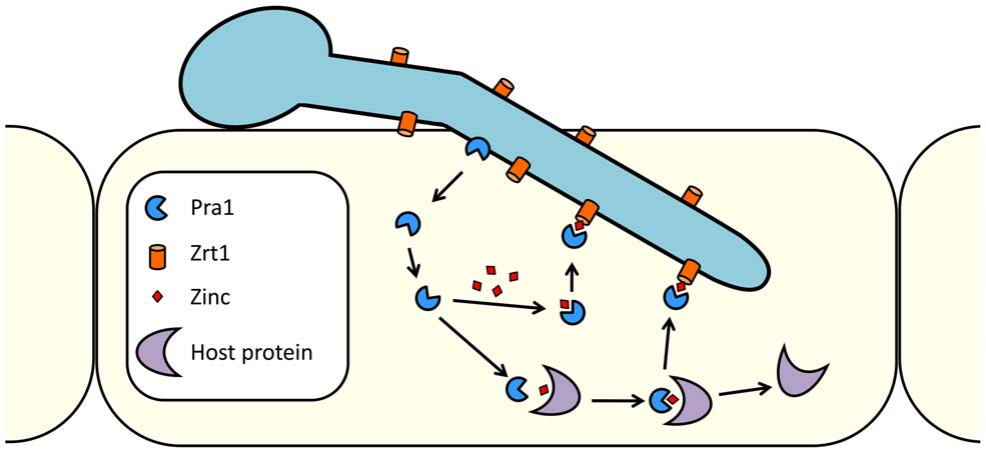
Model of *C. albicans* zinc scavenging from host cells. Following host cell invasion, Pra1 is expressed and secreted. The released fraction of Pra1 binds zinc, either directly from a cellular pool or from host zinc-binding proteins. Reassociation with the cell surface of *C. albicans* is mediated via direct Pra1-Zrt1 interaction.

In the current study we provide evidence that *C. albicans* Pra1 is an extracellular zinc scavenger: (i) invading *C. albicans* hyphae sequestered zinc from endothelial cells (**Figure 1**); (ii) the *C. albicans* secretome hosts a single protein (Pra1) with multiple zinc binding motifs; (iii) the predicted three-dimensional structure of Pra1 accommodates several zinc binding sites (**Figure 2**); (iv) recombinant Pra1 exhibits zinc binding capacity (**Figure 3**); (v) *PRA1* deletion precludes sequestration and utilisation of host zinc by *C. albicans* (**Figure 4**); (vi) *PRA1* is required for host cell damage only in the absence of exogenous zinc (**Figure 5**); (vii) Zrt1 is essential for cellular reassociation of Pra1 (**Figure 9**). Based on these data we propose the following model for *C. albicans* zinc acquisition from the host cell (**Figure 10**). Following host cell invasion, *PRA1* is expressed due to the alkaline pH and low soluble zinc status of the intracellular environment [38], [39], [40], [41]. A fraction of secreted Pra1 is released from the fungal cell surface [32]. This component binds host cellular zinc (either free cytosolic or bound to host protein) and returns to the fungal cell via physical interaction with Zrt1 to deliver its zinc load. Based on this, we suggest the new term “zincophore”, for a secreted zinc binding protein which can sequester this metal from the environment and reassociate with the microbial cell.

In *C. albicans*, Pra1 interaction with components of the innate immune system may be related to its zinc-scavenging function. We note that the cofactor activities of both C4b-binding protein and factor H are zinc dependent [42] and that factor H binds zinc at multiple locations on its surface [43]. It is tempting to speculate that interactions between Pra1 and these complement regulators are zinc mediated. In line with this concept, it is noteworthy that the pneumococcal histidine triad protein of *Streptococcus pneumoniae* has both zinc binding and factor H recruitment properties [44], [45].

Although it is clear that Pra1 plays important immunomodulatory roles, we propose that Pra1 has an evolutionary older biological function, which has remained elusive until now. This function is zinc acquisition. This is based on phylogenetic analyses: *PRA1* orthologues were identified in a number of fungal species which do not associate with vertebrate hosts in nature (**Figure S4**). Moreover, we identified *PRA1* orthologues in both ascomycetes and basidiomycetes, groups which diverged at least 452 million years ago [24]. Indeed, the presence of a *PRA1* orthologue in the basal chytrid, *S. punctatus*, suggests that the origin of this gene may be even older. However, the accuracy of this assessment is limited by the low numbers of sequenced basal fungi. The relatively high sequence identity of *PRA1* orthologues (>30% at the amino acid level) across such distantly related fungal species suggests that the gene is under positive evolutionary selection. Furthermore, conservation of *PRA1* and *ZRT1* synteny between the Ascomycota and Basidiomycota is indicative of an ancient and highly successful evolutionary adaptation.

It is likely that *PRA1*-*ZRT1* modularity simplifies the regulation of these two genes. Indeed, it would appear that transcriptional circuitry is also conserved. In *C. albicans*, *PRA1*/*ZRT1* is regulated by environmental pH and zinc availability via Rim101 and Zap1, respectively (this study, [17], [46]). In *A. fumigatus*, the orthologous gene pair, *aspf2*/*zrfC*, is similarly regulated by pH and zinc via PacC (the Rim101 orthologue) and ZafC (the Zap1 orthologue), respectively [22]. It remains to be determined whether the orthologous zinc scavenger/transporter loci in more distantly related fungal species (e.g. basidiomycetes) are also pH regulated. As bio-available zinc is less soluble at alkaline pH, coupling the regulation of zinc acquisition systems to environmental pH-sensing pathways may be evolutionarily conserved.

Despite the conservation of this zinc scavenging system throughout the fungal kingdom, it has been lost multiple times during evolution (**Figure 8**). As Pra1 serves as the major ligand for leukocyte integrin αMβ2 [32], its loss from the proteomes of human pathogenic fungi (such as *Paracoccidioides brasiliensis*, *Cryptococcus neoformans*, *Histoplasma capsulatum*, *C. glabrata*, *C. lusitaniae* and *C. parapsilosis*) may partially contribute to immune evasion by these species.

However, a clear correlation between retention/loss of *PRA1* and ecological niche is not obviously apparent, and the dynamic loss of *PRA1* orthologues from the genomes of many modern fungal species suggests that these species rely on alternative uptake systems. Indeed, given the essential nature of zinc, it is unlikely that fungi rely solely on one single acquisition system. It is possible that other (Pra1^−^) fungi rely solely on zinc transporters for uptake of this metal from their environment. Indeed, fungal zinc transporters fall into two relatively distinct phylogenetic clusters: those related to *C. albicans ZRT1*, and a second class, related to *C. albicans ZRT2* (**Figure S5**). Alternatively, a different zinc-binding protein may be employed: in principle, another secreted protein with zinc binding properties has the potential to function similarly to Pra1. Finally, some fungi may synthesise secondary metabolites to capture environmental zinc.

Although this is, to our knowledge, the first description of a microbial zinc scavenger (“zincophore”), the Pra1-Zrt1 system shares some functional similarities with bacterial zinc ABC transporters. In both cases, the genes encoding an extracellular zinc binding protein (Pra1 in fungi, ZnuA in bacteria) and zinc transporter (Zrt1 in fungi, ZnuB in bacteria) are encoded at the same locus – bacteria additionally encode an ATP-binding protein (ZnuC) [26]. In prokaryotic ABC import systems, the periplasmic or membrane-associated binding protein (ZnuA) directs its substrate to the permease (ZnuB), thus facilitating import via ATP-hydrolysis (ZnuC) [27]. Similarly, the extracellular zinc-binding protein of *C. albicans*, Pra1, reassociates with the fungal cell via interaction with the transporter, Zrt1 (**Figure 9**). Therefore, our data indicate that syntenic zinc acquisition loci, encoding an extracellular zinc binding protein and a membrane transporter, have been selected twice during evolution.

Future studies on the *C. albicans* zinc acquisition system (or its counterpart in other fungi) will be required to further elucidate details of the Pra1-Zrt1 interaction. For example, our own three-dimensional modelling approach indicates that Pra1-Zrt1 may interact via histidine residues, possibly through the formation of zinc bridges. Therefore, it is possible that zinc-loaded Pra1 binds to Zrt1 with higher affinity than apo-Pra1. Such a simple mechanism may enhance zinc delivery to the fungal cell and may even facilitate release of Pra1 for multiple rounds of zinc scavenging.

## Methods

### Media and growth conditions

*C. albicans* strains were routinely grown in YPD (1% yeast extract, 2% Bacto peptone, 2% glucose). Transformants were selected on SD medium (0.17% Difco yeast nitrogen base; 0.5% ammonium sulphate, 2% glucose, 2% Oxoid agar) supplemented with appropriate amino acids and/or uridine. For zinc starvation, cells were grown in limited zinc medium (LZM). LZM was prepared as described previously [47] with the following modification: zinc was omitted and FeCl_2_ was included at 25 µM. LZM was supplemented with ZnSO_4_ as indicated. For alkaline-induced gene expression, Lee's medium, buffered to pH 5.5 or pH 7.4 was prepared as described previously [48]. For zinc-limited cell culture medium Dulbecco's Modified Eagles Medium (DMEM, PAA, or, where stated Promocell Endothelial growth medium, Promocell) was stripped of metals by incubating with 10% w/v CHELEX-100 beads (Sigma) overnight at 4°C with shaking. All metals with the exception of zinc were then restored to the following concentrations: NaCl 4.4 mg/l, MgSO_4_ 97.7 mg/l, NaHCO_3_ 3.7 mg/l, CaCl_2_ 265 mg/l, KCl 400 mg/l, NaH_2_PO_4_ 109 mg/l. ZnSO_4_ was supplemented as indicated.

### Plasmid and strain construction

*C. albicans* strains used in this study are listed in **Table S1**. Deletion strains produced in this study were generated in the BWP17 background [49] as described previously [50], [51]. A *P_PRA1_*-GFP reporter was generated by amplifying the *PRA1* promoter and cloning into pGFP [16] at *Xho*I and *Hind*III sites. All strains were verified by colony PCR. Primers used for mutant production and verification are listed in **Table S2**. Complementation plasmids were generated by amplifying the gene of interest, including the upstream and downstream intergenic regions followed by cloning into CIp10 [52]. Resultant complementation constructs were linearised with *Stu*I and transformed into strains as stated above.

### Endothelial infection models

Endothelial cells (human umbilical cord endothelial cells – HUVEC) were maintained as described previously [53]. For short term (3.5 h) infections, 10^5^ endothelial cells were seeded on glass cover-slips in 24 well plates and grown for 2 days. Monolayers were washed 3 times with PBS and the medium replaced with zinc-depleted DMEM cell culture medium supplemented with indicated concentration of ZnSO_4_. Monolayers were infected with 10^5^ zinc-pre-starved *C. albicans* cells (via 3 subpassages in LZM at 30°C) for 3.5 h. For long term damage assays, 2×10^4^ cells were seeded into 96 well plates and grown for 2 days. Monolayers were washed 3 times with PBS and medium replaced with zinc depleted DMEM+1% fetal bovine serum (FBS, PAA) supplemented with indicated concentration of ZnSO_4_. Monolayers were infected with 5×10^4^ zinc-starved *C. albicans* cells. At 24 h, supernatant was assayed for lactate dehydrogenase (LDH) release as described previously [54]. For micro-colony experiments, endothelial cells were grown in Promocell endothelial growth medium (Promocell), seeded into 24 well plates and grown for 2 days. Monolayers were washed 3 times with PBS, the medium replaced with zinc-depleted cell culture medium supplemented with 20 µM ZnSO_4_ or 8 µM EGTA and infected with around 50 *C. albicans* cells. For these experiments, *C. albicans* strains were pre-grown for 24 h in YPD to ensure the presence of single cells for inoculation. Following 16 h incubation, micro-colonies were photographed using a Leica DMIL inverse microscope.

### Visualisation of zinc content of endothelial-associated hyphae

Endothelial monolayers, infected with *C. albicans*, were washed 2 times with CHELEX-100-treated PBS and fixed with Roti-Histofix 4% for 30 minutes at room temperature. In subsequent steps, samples were washed 3 times with CHELEX-100-treated PBS between each step and every treatment was performed in CHELEX-100-treated PBS. The extracellular (non-invading) *C. albicans* cells stained with rat anti-*C. albicans* Mab CA-1 (Acris antibodies) (dilution 1/2000) at 30°C 130 rpm. *C. albicans* cells were then counterstained with secondary Mab anti-rat IgM conjugated with Alexa 467 (dilution 1/5000) at 37°C. The endothelial cells were then permeabilized in 0.5% Triton X-100. Entire *C. albicans* cells (extracellular and intracellular) were then stained for 20 min with Concanavalin-A-488 (Molecular Probes) (17 µg/ml). Labile zinc was then stained with 12 µM zinquin (Sigma) for 3 h at 30°C and 80 rpm. The coverslips were mounted inverted on a microscope slide, with ProLong Gold Antifade Reagent and observed under epifluorescence using filters to detect Alexa-467, Alexa-488 and DAPI (for zinquin).

Invading *C. albicans* cells were determined as previously described [54]. Hyphal length was determined using Leica Application Suite software. Hyphal-zinc localisation was determined by selecting the relevant section (either the entire hypha, extracellular section only or invading section only) of each filament using Leica Application Suite AF selection tool and measuring fluorescence. The fluorescence values were then normalised per 0.5 µm^2^. The experiment was performed twice in triplicate and at least 25 cells were measured (both for length and fluorescence) per replicate (<150 cells per sample).

### *In silico* analysis and three-dimensional modelling

The amino acid sequences of all 55 confirmed and predicted *C. albicans* secreted proteins (GO term: extracellular region, *Candida* Genome Database) were analysed using protein-pattern-find (bioinformatics.org/sms2/protein_pattern) for the presence of zinc binding motifs [11]. Three-dimensional models of both Pra1 and Zrt1 were generated using Phyre^2^ (www.sbg.bio.ic.ac.uk/phyre2). Only sections of the protein which were modelled with a confidence of 30% or more are displayed in the predicted structures. The Pra1 best structural hit (Deuterolysin from *A. oryzae*) was additionally identified using SWISS-MODEL tool (swissmodel.expasy.org) [55] and metal binding sites predicted with Findsite-metal (cssb.biology.gatech.edu/findsite-metal) [56] and 3D ligand site (www.sbg.bio.ic.ac.uk/3dligandsite). To predict interactions between the two proteins, the structures generated in Phyre^2^ were analysed with Patchdock (bioinfo3d.cs.tau.ac.il/PatchDock) [57]. To confirm the predicted interaction shown in **Figure 9**, PepSite (pepsite.russelllab.org/index.html) [58] was used. Briefly, the C-terminal tail of Pra1 was divided into 10 amino acid segments and tested for interaction with the three-dimensional model of Zrt1. Reciprocally, the N-terminal extracellular tail of Zrt1 (defined by TMHMM - www.cbs.dtu.dk/services/TMHMM) was divided into 10 amino acid peptides and tested for interaction with the three-dimensional model of Pra1.

### Protease assay

Proteolytic activity of recombinant proteins against casein was tested with the Enzchek Protease Assay Kit (Molecular Probes). Briefly, BODIPY FL casein (10 µg/ml) was incubated with 0.5 µg recombinant Pra1 or thermolysin (Sigma) in 50 mM Tris-HCl pH 8 at 37°C and fluorescence was measured at 485 nm excitation/525 nm emission. Selected samples contained the metalloprotease inhibitor phenanthroline (10 mM).

### Determination of Pra1 zinc-binding

Equal amounts (0.4 nmol) of either recombinantly-expressed His-tagged Pra1 [13] and His-tagged β-galactosidase [14] were loaded onto 10 kDa microspin columns (Ambion), washed twice with 500 µl HS buffer (50 mM HEPES-KOH pH 7.5, 200 mM NaCl), transferred to reaction tubes and incubated with 0.1 mM ZnSO_4_ for 1 h at 30°C. The zinc-loaded proteins were then transferred to 10 kDa microspin columns and sequentially washed with 150 µl of HS buffer. Each flow-through was assayed for zinc content by PAR assay as described previously [59]. Briefly, 4-(2-pyridylazo)resorcinol (PAR) was added to each sample to 0.1 mM and optical density measured at 490 nm against a ZnSO_4_ standard curve. Following 30 washes, undigested and digested (40 µg proteinase K, 60°C for 30 min) samples were again assayed for zinc content by PAR assay.

### *PRA1* expression

*C. albicans* strains carrying empty vector, *P_ACT1_-GFP* [60] or *P_PRA1_*-GFP were grown overnight in non-buffered Lee's medium [48] and diluted to OD_600_ = 0.05 in Lee's medium buffered to either 5.5 (MES) or 7.4 (HEPES), containing standard (3.7 µM) or high (100 µM) ZnSO_4_. Cell suspensions were incubated in flat, transparent-bottomed, black-walled 96 well plates (Nunclon) in a Magellan TECAN plate reader with 30 s shaking and fluorescence (484/525 nm) measurements taken every 15 min. For each sample condition and time point, auto-fluorescence determined from *C. albicans* carrying the empty vector was subtracted from *P_PRA1_*-GFP and *P_ACT1_-GFP* values, yielding normalised fluorescence values. For clarity, measurements taken at 2 h intervals were plotted.

### Limited zinc growth curves

Strains were pre-grown for 2 days at 30°C in unbuffered LZM, without additional zinc. Cells were diluted to OD_600_ = 0.05 in LZM, buffered to pH 7.4 (HEPES) supplemented with indicated concentrations of ZnSO_4_. Cultures were incubated at 37°C in a Magellan TECAN plate reader with 30 s shaking and OD_600_ determined every 15 min. For clarity, measurements taken at 2 h intervals were plotted.

### Binding of recombinant Pra1 to *C. albicans*

For microscopic evaluation of Pra1 binding to *C. albicans*, 10^6^ cells from an LZM overnight preculture were seeded onto coverslips in 24 well tissue culture plates in 1 ml RPMI 1640 (PAA)+10% FBS and incubated for 3 h at 37°C, 5% CO_2_. Coverslips were washed once with PBS and incubated for 1 h in 500 µl PBS with 1% bovine serum albumin (BSA) and 60 µg/ml recombinant His-tagged Pra1 [13]. Coverslips were then washed three times with PBS to remove non-bound rPra1 and fixed with 500 µl Roti-Histofix 4%. Following fixation, coverslips were washed three times with PBS and incubated with rabbit anti-His FITC-conjugated antibody (Abcam) (diluted 1∶200 in PBS with 1% BSA) for 1 h at room temperature. Coverslips were then washed three times with PBS and mounted inverted onto microscope slides using ProLong Gold Antifade Reagent (Invitrogen). At least 50 cells per sample were photographed with a Leica DM 5500B microscope (Leica) using the filter set to detect FITC.

For Western blot evaluation of Pra1 binding to *C. albicans*, strains were grown overnight in Lee's medium buffered to pH 7.4 at 37°C. Cells were then washed once with PBS, adjusted to 5×10^8^ cells/ml in PBS with 40 µg/ml recombinant His-tagged Pra1 and incubated for 1 h at 37°C. Cells were then washed 3 times with PBS to remove unbound rPra1. Total protein was extracted by resuspending cells in 200 µl PBS containing 3 mM KCl, 2.5 mM MgCl_2_, 0.1% TritonX-100 and protease inhibitor cocktail (Roche) in screw-cap reaction tubes containing 10% w/v glass beads. Cells were lysed in a bead mill (Precellys) for 2 braking cycles of 30 s. at 2800× g. Samples were then centrifuged for 10 min at 18900× g at 4°C. The lysate was transferred to new reaction tubes and protein content quantified with DC protein assay (Biorad). Forty µg of protein extract of each strain were separated in 12% Tris-Glycine SDS-PAGE and blotted onto nitrocellulose membrane. The membrane was blocked in PBS+0.05% Tween-20 (PBST) containing 2% milk powder and 1% BSA for 60 min at room temperature. The membrane was hybridised with rabbit anti-His-tag antibody (Abcam) (dilution 1∶500) in PBST containing 2% milk powder overnight at 4°C. The membrane was then washed 3 times with PBST for 5 min at room temperature and hybridized with secondary anti-rabbit-immunoglobulin-HRP-conjugated antibody (Dako), (dilution 1∶2000) in PBST containing 2% milk powder for 2 h at room temperature. After 3 washes with PBST for 5 min at room temperature and an additional PBS wash of 1 min, bands were identify by Enhanced Chemiluminescence (ECL) using the SuperSignal West Dura kit (Thermo Scientific). The membrane was then stripped according to a standard protocol and re-hybridised with rat anti-α tubulin antibody (AbD Serotec) (dilution 1∶2000) and anti-rat-immunoglobulin-HRP-conjugated antibody (Dako), (dilution 1∶2000) as described above. The experiment was performed twice with comparable results.

### Identification of Pra1 orthologues

The *C. albicans* Pra1 sequence (*Candida* Genome Database) was analysed by BLASTp at the NCBI (blast.ncbi.nlm.nih.gov/Blast.cgi) and BROAD (www.broadinstitute.org) databases. Identified orthologues were analysed with MegAlign and aligned with ClustalW, Gonnet series protein weight matrix. Resulting alignment was displayed as a phylogram. Additionally, *C. albicans* Zrt1 and Zrt2 orthologues were identified by BLASTp at NCBI and BROAD. Selected best hits were aligned with MegAlign.

Syntenic arrangements of fungal *PRA1* and *ZRT1* orthologues were investigated at the BROAD institute database, yeast gene order browser [61], *Candida* gene order browser [62], the *Candida* Genome database or the *Aspergillus* Genome database (www.aspgd.org). Bacterial zinc transporter encoding genes were investigated at the PathoSystems Resource Integration Center (www.patricbrc.org).

## Supporting Information

Figure S1 **Pra1 does not exhibit proteolytic activity.** Recombinant Pra1 or thermolysin were incubated with BODIPY FL casein and fluorescence at 485/525 nm measured at indicated time points. Experiment was performed twice.
(TIF)

Figure S2 ***ZRT1*** **is required for growth under zinc depletion.** *C. albicans* wild type (M1477), *zrt1*Δ (M2006) or *zrt1*Δ+*ZRT1* (M2010) strains were grown overnight in LZM medium and used to inoculate LZM medium, buffered to pH 7.4 containing: no additional zinc (**A**), 0.5 µM (**B**) or 20 µM (**C**) additional zinc. Cultures were incubated in a Tecan plate reader and optical density at 600 nm measured at indicated time points. Experiment was performed twice.
(TIF)

Figure S3 ***ZRT1*** **and** ***PRA1*** **are required for microcolony development on endothelia in the absence of exogenous zinc.** Single cells of *C. albicans* wild type (M1477), *zrt1*Δ (M2006), *zrt1*Δ+*ZRT1* (M2010), *pra1*Δ (M2008) or *pra1*Δ+*PRA1* (M2012) were incubated for 16 h in either zinc-replete (+Zn) or zinc-depleted (+EGTA) cell culture medium on either endothelial monolayers (Endo) or on plastic (Ctrl). Note that *pra1*Δ and *zrt1*Δ only formed microcolonies in the presence of exogenously added zinc whereas wild type and complemented strains were capable of microcolony development on endothelial monolayers in the absence of exogenous zinc. Experiment was performed three times. Representative images are shown.
(TIF)

Figure S4 **Phylogeny of Pra1 orthologues throughout the fungal kingdom.** All available amino acid sequences of Pra1 orthologues (NCBI and BROAD databases) were aligned using ClustalW. (**A**) Phylogram of Pra1 alignment. Selected lineages are highlighted (arrows). Note that, where present, Pra1 sequence similarity agrees well with overall species phylogeny. (**B**) Alignment of conserved predicted zinc binding motifs (see Figure 2). *C. albicans* is highlighted with a red asterisk. The positions of the histidine residues in the *C. albicans* sequence are marked with red dots above the alignment. Note that HAXXHXL (positions 68–74) is specific to ascomycetes and that basidiomycetes encode HNSIXHXL at the same position. Both HAXXHXL and His^193^ have been lost by WGD clade yeast. The HRXXH motif (positions 178–182) is fully conserved in all known Pra1 orthologues.
(TIF)

Figure S5 **Phylogeny of fungal zinc transporter-encoding genes.** *C. albicans* Zrt1 and Zrt2 amino acid sequence best hits in selected fungal species were aligned using ClustalV and plotted as a phylogram. *S. cerevisiae* Zrt1 was also included for comparison. Cognate encoding genes which are syntenic with *PRA1* orthologues are indicated with red asterisks. Note that fungal zinc transporters fall into two general classes – those related to *C. albicans* Zrt1 and those related to *C. albicans* Zrt2 – and that only direct orthologues of *C. albicans ZRT1* are syntenic with *PRA1* orthologues.
(TIF)

Table S1 **Strains used in this study.** Genotype and sources for all *C. albicans* strains used in this study.
(DOC)

Table S2 **Primers used in this study.** Name, sequence and purpose of each primer used in this study. Bold indicates annealing sites to pFA plasmids [50]. Underlined basepairs indicate added restriction sites; * are from [50].
(DOC)
