# Supplementary figures and images for: Candida albicans Scavenges Host Zinc via Pra1 during Endothelial Invasion
Source: PLoS Pathog. 2012 Jun 28;8(6):e1002777. doi: 10.1371/journal.ppat.1002777 (PMC3386192; doi:10.1371/journal.ppat.1002777)

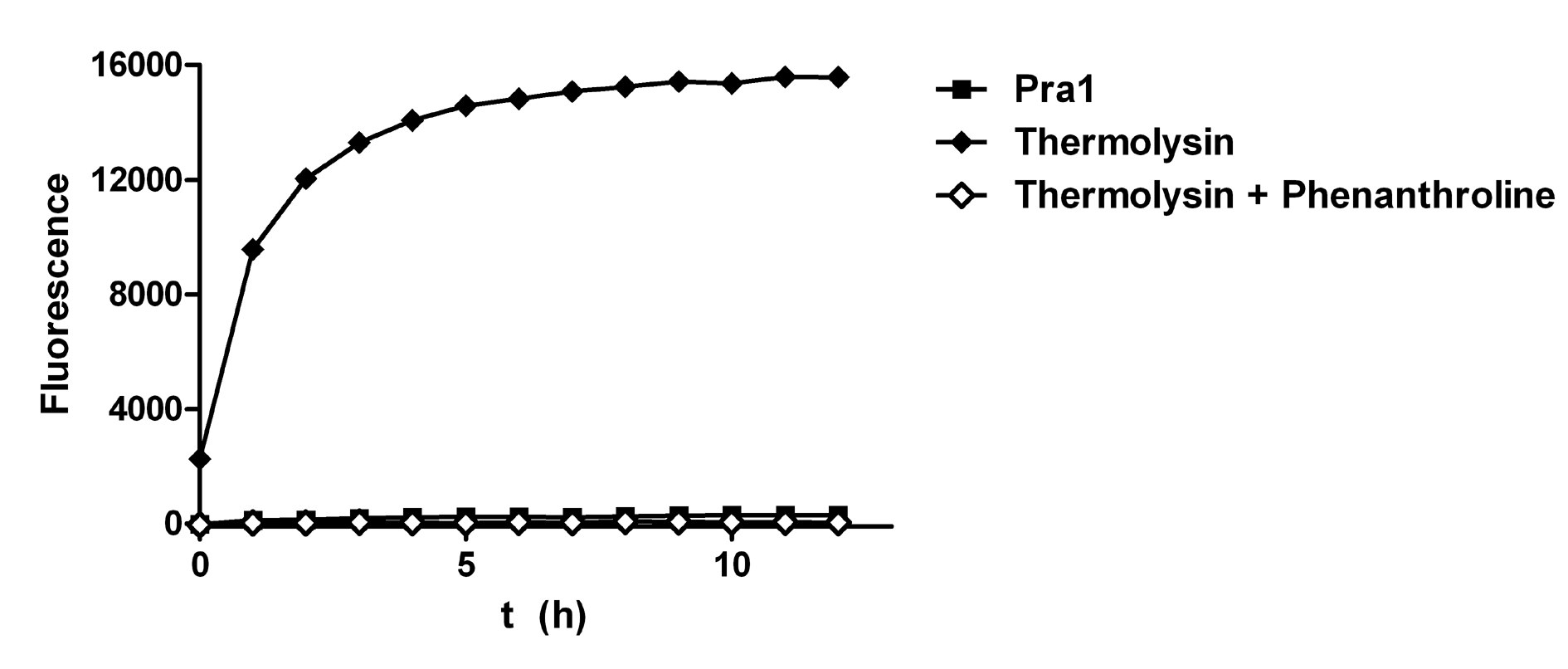

Supplement: Figure S1 — Pra1 does not exhibit proteolytic activity. Recombinant Pra1 or thermolysin were incubated with BODIPY FL casein and fluorescence at 485/525 nm measured at indicated time points. Experiment was performed twice. (TIF) [file ppat.1002777.s001.tif]

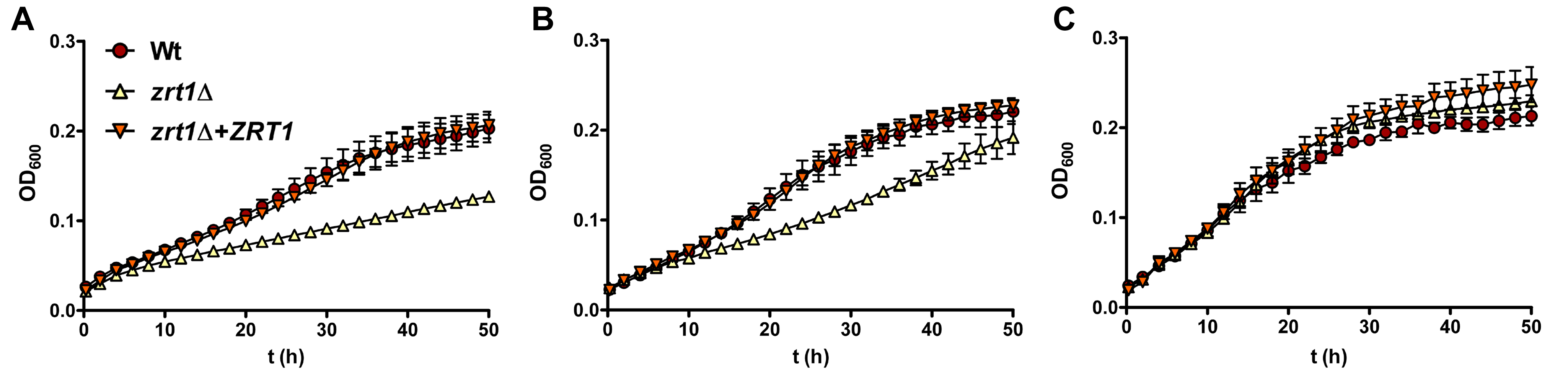

Supplement: Figure S2 — ZRT1 is required for growth under zinc depletion. C. albicans wild type (M1477), zrt1Δ (M2006) or zrt1Δ+ZRT1 (M2010) strains were grown overnight in LZM medium and used to inoculate LZM medium, buffered to pH 7.4 containing: no additional zinc (A), 0.5 µM (B) or 20 µM (C) additional zinc. Cultures were incubated in a Tecan plate reader and optical density at 600 nm measured at indicated time points. Experiment was performed twice. (TIF) [file ppat.1002777.s002.tif]

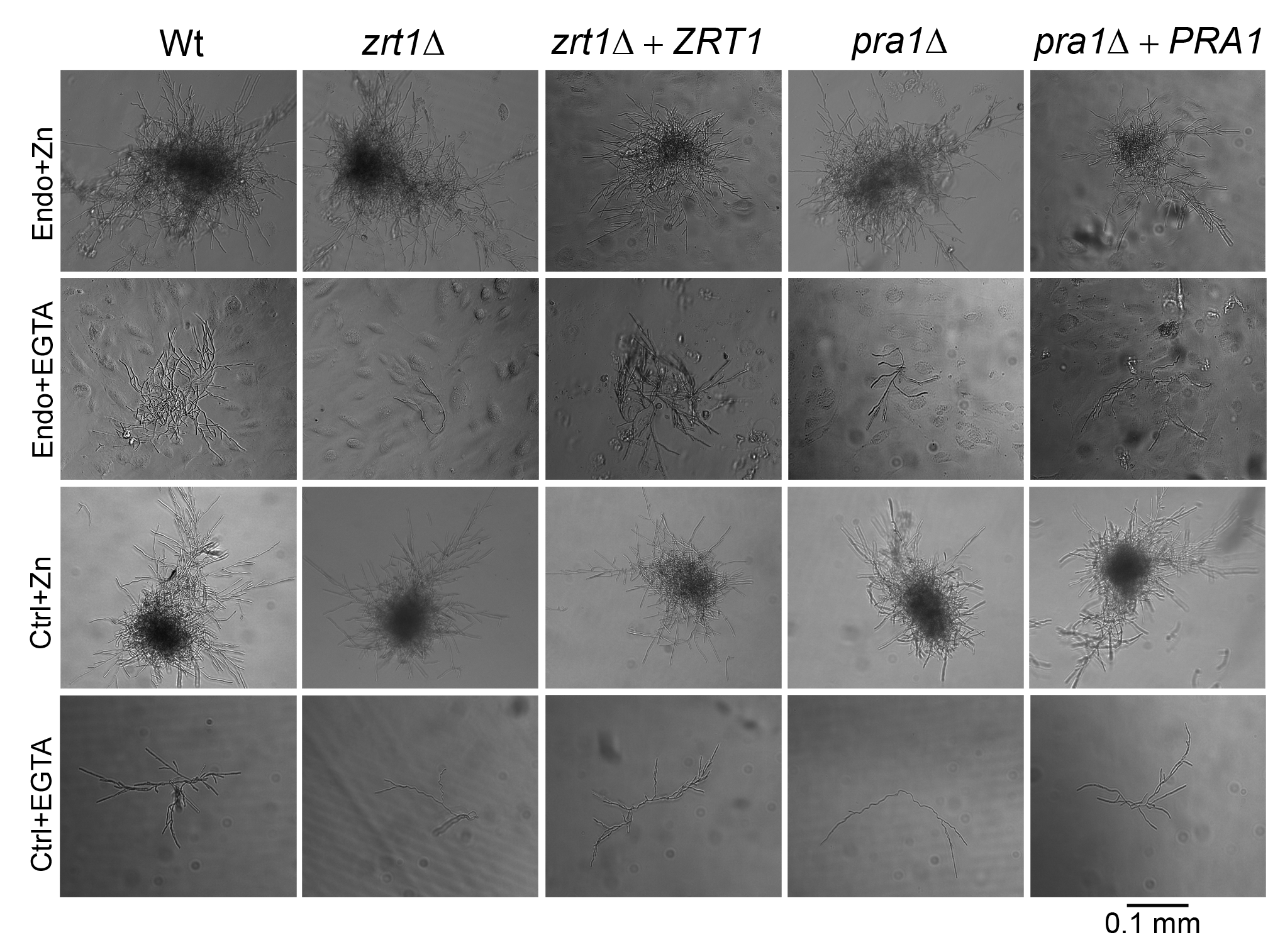

Supplement: Figure S3 — ZRT1 and PRA1 are required for microcolony development on endothelia in the absence of exogenous zinc. Single cells of C. albicans wild type (M1477), zrt1Δ (M2006), zrt1Δ+ZRT1 (M2010), pra1Δ (M2008) or pra1Δ+PRA1 (M2012) were incubated for 16 h in either zinc-replete (+Zn) or zinc-depleted (+EGTA) cell culture medium on either endothelial monolayers (Endo) or on plastic (Ctrl). Note that pra1Δ and zrt1Δ only formed microcolonies in the presence of exogenously added zinc whereas wild type and complemented strains were capable of microcolony development on endothelial monolayers in the absence of exogenous zinc. Experiment was performed three times. Representative images are shown. (TIF) [file ppat.1002777.s003.tif]

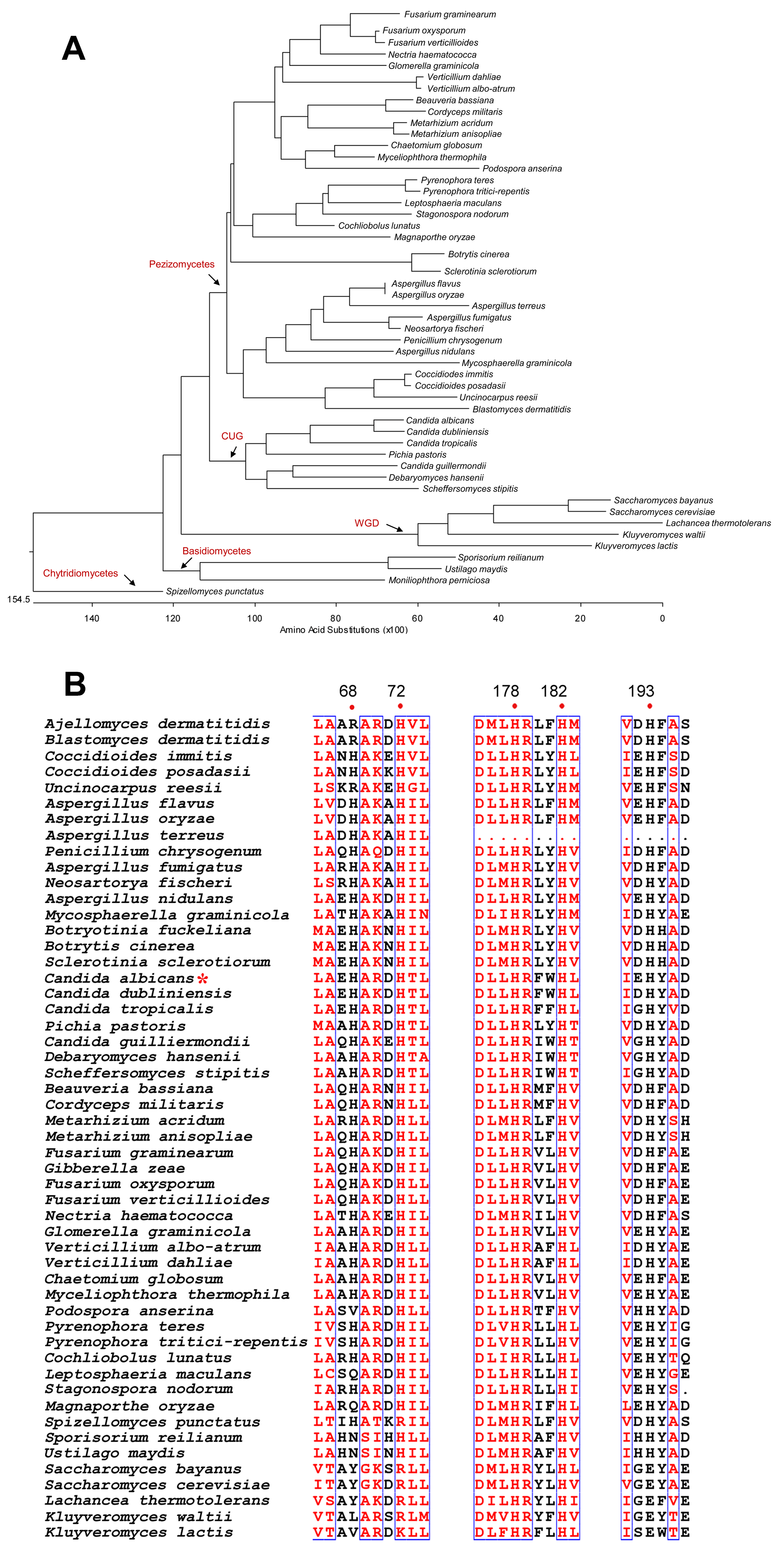

Supplement: Figure S4 — Phylogeny of Pra1 orthologues throughout the fungal kingdom. All available amino acid sequences of Pra1 orthologues (NCBI and BROAD databases) were aligned using ClustalW. (A) Phylogram of Pra1 alignment. Selected lineages are highlighted (arrows). Note that, where present, Pra1 sequence similarity agrees well with overall species phylogeny. (B) Alignment of conserved predicted zinc binding motifs (see Figure 2). C. albicans is highlighted with a red asterisk. The positions of the histidine residues in the C. albicans sequence are marked with red dots above the alignment. Note that HAXXHXL (positions 68–74) is specific to ascomycetes and that basidiomycetes encode HNSIXHXL at the same position. Both HAXXHXL and His193 have been lost by WGD clade yeast. The HRXXH motif (positions 178–182) is fully conserved in all known Pra1 orthologues. (TIF) [file ppat.1002777.s004.tif]

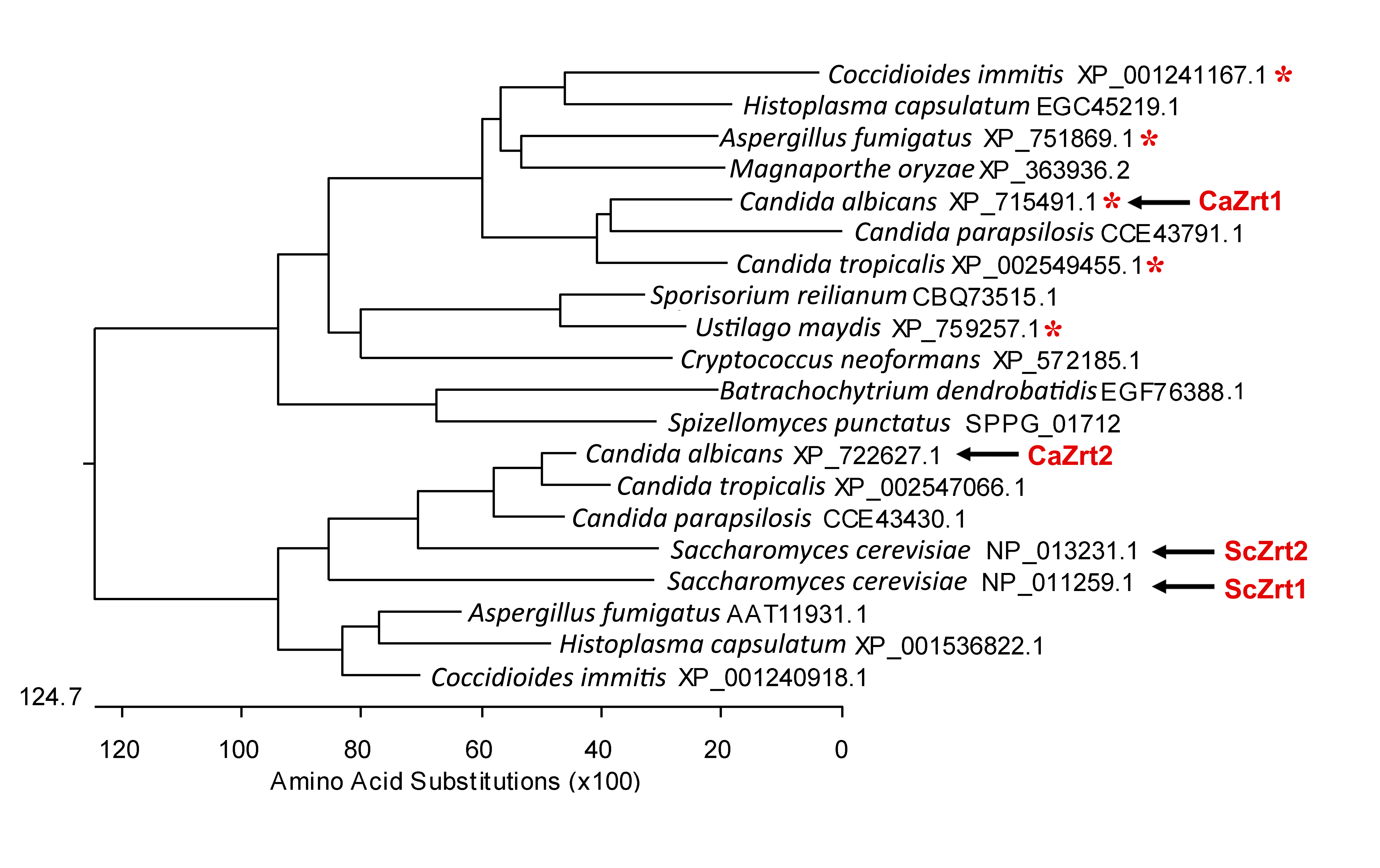

Supplement: Figure S5 — Phylogeny of fungal zinc transporter-encoding genes. C. albicans Zrt1 and Zrt2 amino acid sequence best hits in selected fungal species were aligned using ClustalV and plotted as a phylogram. S. cerevisiae Zrt1 was also included for comparison. Cognate encoding genes which are syntenic with PRA1 orthologues are indicated with red asterisks. Note that fungal zinc transporters fall into two general classes – those related to C. albicans Zrt1 and those related to C. albicans Zrt2 – and that only direct orthologues of C. albicans ZRT1 are syntenic with PRA1 orthologues. (TIF) [file ppat.1002777.s005.tif]
