# Supplementary material for: Candida albicans Scavenges Host Zinc via Pra1 during Endothelial Invasion
Source: PLoS Pathog. 2012 Jun 28;8(6):e1002777. doi: 10.1371/journal.ppat.1002777 (PMC3386192; doi:10.1371/journal.ppat.1002777)
Supplement: Table S1 — Strains used in this study. Genotype and sources for all C. albicans strains used in this study. (DOC) [file ppat.1002777.s006.doc]

| Strain | Name | Genotype | Reference |
| --- | --- | --- | --- |
| M134 | Wild type  CAI-4 + CIp10 | ura3::imm434 / ura3::imm434 + CIp10 | [60] |
| M1784  (CAMB435) | *pra1*∆ ura^-^ | ura3::imm434/ura3::imm434 pra1::hisG/pra1::hisG | [15] |
| M1809 | *pra1*∆ | ura3::imm434/ura3::imm434 pra1::hisG/pra1::hisG + CIp10 | This study |
| M1785  (CAMB9) | *pra1*∆ + *PRA1* | ura3::imm434/ura3::imm434 pra1::hisG/pra1::PRA1-pUC18-UR | [15] |
| M1522 | *P_PRA1_*-GFP | ura3::imm434/ura3::imm434  +CIp10- *P_PRA1_*-GFP | This study |
| M137 | *P_ACT1_*-GFP | ura3::imm434/ura3::imm434  +CIp10- *P_ACT1_*-GFP | [60] |
| M1477 | Wild type  BWP17 +CIp30 | ura3:: imm434/ura3:: imm434 his1::hisG/his1::hisG + CIp30 | This study |
| M130 | BWP17 | ura3:: imm434/ura3:: imm434 his1::hisG/his1::hisG arg4::hisG/arg4::hisG | [49] |
| M1970 | pra1h | ura3:: imm434/ura3:: imm434 his1::hisG/his1::hisG *pra1*::*HIS1*/*PRA1* | This study |
| M2005 | pra1ha | ura3:: imm434/ura3:: imm434 his1::hisG/his1::hisG *pra1*::*HIS1*/*pra1*::*ARG4* | This study |
| M2008 | *pra1*∆ | ura3:: imm434/ura3:: imm434 his1::hisG/his1::hisG *pra1*::*HIS1*/*pra1*::*ARG4* +CIp10 | This study |
| M2012 | *pra1*∆+*PRA1* | ura3:: imm434/ura3:: imm434 his1::hisG/his1::hisG *pra1*::*HIS1*/*pra1*::*ARG4* +CIp10-*PRA1* | This study |
| M1973 | zrt1h | ura3:: imm434/ura3:: imm434 his1::hisG/his1::hisG *zrt1*::*HIS1*/*ZRT1* | This study |
| M2004 | zrt1ha | ura3:: imm434/ura3:: imm434 his1::hisG/his1::hisG *zrt1*::*HIS1*/*zrt1*::*ARG4* | This study |
| M2006 | *zrt1*∆ | ura3:: imm434/ura3:: imm434 his1::hisG/his1::hisG *zrt1*::*HIS1*/*zrt1*::*ARG4* +CIp10 | This study |
| M2010 | *zrt1*∆+*ZRT1* | ura3:: imm434/ura3:: imm434 his1::hisG/his1::hisG *zrt1*::*HIS1*/*zrt1*::*ARG4* +CIp10-*ZRT1* | This study |
