# Supplementary material for: Candida albicans Scavenges Host Zinc via Pra1 during Endothelial Invasion
Source: PLoS Pathog. 2012 Jun 28;8(6):e1002777. doi: 10.1371/journal.ppat.1002777 (PMC3386192; doi:10.1371/journal.ppat.1002777)
Supplement: Table S2 — Primers used in this study. Name, sequence and purpose of each primer used in this study. Bold indicates annealing sites to pFA plasmids [50]. Underlined basepairs indicate added restriction sites; * are from [50]. (DOC) [file ppat.1002777.s007.doc]

| **Name** | **Sequence 5->3’** | **Purpose** |
| --- | --- | --- |
| ZRT1-FG | TCTCTTGGCGACCGATGGAATCTTGGAAAACGGAATAATTAATATAAAGGTAGCCTTTGCTGAATATGTTTTTGGTGGTAGCTCTCTAAAAATTAACAACATCA**GAAGCTTCGTACGCTGCAGGTC** | Generation of *ZRT1* deletion construct |
| ZRT1-RG | GGTGGTCAGAGGCATTGGTCGCCAAACTTCAAACCAATAAGTTGGTAATTTACAGCTGCATATATTAGTATGTTCTGTAGTAATGTATATATGTATACGGAAAT**TCTGATATCATCGATGAATTCGAG** | Generation of *ZRT1* deletion construct |
| ZRT1-F1 | GAGGTTGCCCAGAATAGTTGG | Confirmation of ZRT1 deletion |
| ZRT1-R1 | TCAAGGCCGTTGAGAAAGAATACG | Confirmation of ZRT1 deletion |
| ZRT1-Rec-F1 | gctGTCGACTGACATACTCAAAGCCAATCACAG | Construction of *ZRT1* complementation plasmid |
| ZRT1-Rec-R1 | GAATACGCGTCCATGAAGATACCGAG | Construction of *ZRT1* complementation plasmid |
| PRA1-FG | TTAACCAAAGTATAAAGAGGCAACAATATCTCGTTGGAAAAGACCTTTGTTTGGTTAATCATTTTTTTTATTCACATCTATAATCACAAACTTTCTCTCGAAAT**GAAGCTTCGTACGCTGCAGGTC** | Generation of *PRA1* deletion construct |
| PRA1-RG | TACTCTTTTGTCTACATACGATTTTGCAATTAATCTTATTAATTCAAGCTATAAAAGATATCCATGAAACACCTTAAAAATTGTTTAATGCCTGAACTTAACAA**TCTGATATCATCGATGAATTCGAG** | Generation of *PRA1* deletion construct |
| PRA1-F1 | CGCACCCACGCACTCTCATC | Confirmation of *PRA1* deletion |
| PRA1-R1 | TGGGAAACCGAGAGGCTTTGTCG | Confirmation of *PRA1* deletion |
| PRA1-Rec-F1 | GCTGTCGACCTACCACCAAAAACATATTCAGC | Construction of *PRA1* complementation plasmid |
| PRA1-Rec-R1 | GCAACGCGTAATGGGCAAATGATTGCTCCTTC | Construction of *PRA1* complementation plasmid |
| PRA1-F2 | GATCCTCGAGATCGACACAATGCTC | Construction of *P_PRA1_*-GFP reporter |
| PRA1-R2 | GATCAAGCTTAGAGAAAGTTTGTGATT | Construction of *P_PRA1_*-GFP reporter |
| PRA1-Inf1 | ACCACACATTGAGATTCGGTAGC | Confirm plasmid integration |
| URA-F2* | GGAGTTGGATTAGATGATAAAGGTGATGG | Confirm plasmid integration |
| RPF-1 | GAGCAGTGTACACACACACATCTTG | Confirm plasmid integration |
| RPF-2 | CGCCAAAGAGTTTCCCCTATTATC | Confirm plasmid integration |
| HIS-F2* | GGACGAATTGAAGAAAGCTGGTGCAACCG | Confirm auxotrophic marker integration |
| HIS-R2* | CAACGAAATGGCCTCCCCTACCACAG | Confirm auxotrophic marker integration |
| ARG-F2* | GGATATGTTGGCTACTGATTTAG | Confirm auxotrophic marker integration |
| ARG-R2* | AATGGATCAGTGGCACCGGTG | Confirm auxotrophic marker integration |

Bold indicates annealing sites to pFA plasmids [50]

Underlined basepairs indicate added restriction sites

* are from [50]
